# Microemulsions and Nanoemulsions for Topical Delivery of Tripeptide-3: From Design of Experiment to Anti-Sebum Efficacy on Facial Skin

**DOI:** 10.3390/pharmaceutics16040554

**Published:** 2024-04-19

**Authors:** Nontachai Magrode, Worrapan Poomanee, Kanokwan Kiattisin, Chadarat Ampasavate

**Affiliations:** 1 Department of Pharmaceutical Sciences, Faculty of Pharmacy, Chiang Mai University, Chiang Mai 50200, Thailand; nontachai_m@cmu.ac.th (N.M.); worrapan.p@cmu.ac.th (W.P.); kanokwan.k@cmu.ac.th (K.K.); 2 Center for Excellence in Pharmaceutical Nanotechnology, Faculty of Pharmacy, Chiang Mai University, Chiang Mai 50200, Thailand

**Keywords:** Tripeptide-3, oil-control, nanoemulsions, microemulsions, anti-sebum, topical, design of experiment, efficacy, dermal delivery

## Abstract

The targeted delivery of a hydrophilic Tripeptide-3 to the skin using microemulsions or nanoemulsions for facial oil reduction was the focus of this study. The impact factors affecting oil/water transparent dispersion formation, such as the surfactant system, HLB value, and co-solvent, were identified through the water titration method and pseudoternary phase diagram plots. The interfacial tension between caprylic/capric triglyceride (CCT oil) and water was significantly reduced by the surfactant/co-surfactant combination (S_mix_) of Cremophore^®^ RH40 and a double-tails co-surfactant, polyglycerol-3-diisostearate, at an HLB of 13 together with a water-to-co-solvent (PG) ratio of 1:1. A two-level full factorial design of experiment (FFD-DoE) emphasized the independent variables of the HLB value, co-solvent, and CCT oil contents affecting the optimal compositions for micro- or nanoemulsion formation. The low-energy spontaneous emulsification of the optimized combination at a low S_mix_ content (10%) yielded the translucent oil-in-water Tripeptide-3 nanoemulsions with an internal droplet size of 25.7 ± 1.20 nm, a narrow polydispersity index of 0.237 ± 0.129, and 70.6 ± 0.58% transmittance. The in vitro skin permeation study revealed a significantly higher skin penetration and retention of the Tripeptide-3 nanoemulsions compared to the high surfactant microemulsions and coarse emulsions. Skin irritation and oil control efficacy were evaluated in healthy volunteers before and after product application for 28 days. The obtained nanoemulsions not only decreased sebum production but also enhanced skin moisture levels. In conclusion, the meticulously designed nanoemulsions, incorporating suitable excipients, show a promising delivery system for hydrophilic peptides to control sebum overproduction in oily facial skin.

## 1. Introduction

Excessive sebum production, prevalent in teenagers and people of all ages, can lead to skin problems such as shiny skin, acne, and blemishes [1,2]. Many people with oily faces and/or severe acne on their skin significantly suffer from these skin problems which impact their quality of life and self-confidence [3]. Many factors can cause the overproduction of sebum (oil), building up dead skin cells, and plugging skin pores, producing blackhead and whitehead acne. Consequently, bacterial infection can exacerbate skin conditions to severely infected acne. Currently, various remedies are available; nevertheless, very few strategies are focused on the origin of acne production [1,2,3]. Intradermal botulinum toxin (Botox^®^) injection is an invasive treatment for excess oil production in severely oily skin. This treatment requires professional licences or a dermatologist to perform the injection [4,5]. Alternatively, the topical application of Botox-like agents has been interesting when incorporated into cosmetics because of their various cosmeceutical functions.

One of the Botox-like peptides, the Tripeptide-3 has been reported to be effective in controlling excess sebum. Acne, oily/shiny skin, and an excess of sebum can be caused by overactive sebaceous glands or sebaceous hyperplasia (enlarged sebaceous glands). Dipeptidyl peptidase IV (DPP4) inhibitors used topically could suppress sebocytes proliferation and function [6]. According to the patent, topical applications of one of the DPP4 inhibitors that included Tripeptide-3 could prevent or treat disorders of the sebaceous glands, including sebaceous hyperplasia, hyperseborrhea, acne, seborrheic dermatitis, atopic dermatitis, and rosacea [6]. 

However, the hydrophilic character of this peptide (LogP value approx. −3) [5] and the natural defense of the stratum corneum (SC) are the main obstacles for the peptide delivery to the target sites, i.e., sebaceous glands in the dermis [7]. Many delivery carriers have been evaluated to improve the skin permeation of anti-wrinkle (Botox-like) peptides such as phospholipid-based vesicular nanocarriers, liquid crystal nanoparticles, or microemulsions [8]. Microemulsions (MEs) and nanoemulsions (NEs) are popular formulations for the delivery of pharmaceuticals and cosmeceuticals. The advantage of these delivery systems is the ease of manufacturing in which spontaneous or low-energy emulsification without complicated instruments can generate efficient delivery systems for both hydrophilic and hydrophobic compounds [9,10]. Visually, MEs and NEs exhibit transparent or translucent liquid textures with different viscosity and rapid absorption due to the vast increase in interfacial areas of formulations. Both MEs and NEs are submicron emulsions including two immiscible liquids, such as oil and water, stabilized by an interfacial film of surfactant molecules, and, if necessary, a co-surfactant and/or co-solvent. Various internal-droplet size ranges of MEs have been reported of 1–100 nm [11] and 5–200 nm [9]. The microstructures of MEs and NEs as oil-in-water (O/W) or water-in-oil (W/O) droplets, and bicontinuous structures, can be formed over a wide range of compositions dependent on the properties of the oil and the surfactant [12]. Mean droplet diameters ranging from 25–500 nm even 1000 nm have been reported for NEs [13]. MEs and NEs were demonstrated to improve skin permeation performance compared to other encapsulation strategies. They exhibited not only the enhanced transdermal ability of the active compound(s) but also the simplicity and scalability of production [8,10,14].

In contrast to MEs which can form spontaneously by gentle stirring, NEs require some energy to reduce the existing free energy through several high- or low-energy methods. By using a low-energy approach, spontaneous emulsification can deliver varieties of transparent or opalescence NEs with reduced thermodynamic stability [15]. Moreover, concerning the production techniques that can differentiate the MEs and NEs, NEs were frequently characterized based on the lower amount of surfactants required, resulting in a lower viscosity of the ultra-fine colloidal dispersion. Moreover, the kinetic stability and dilution ability of the NEs prevent or delay conventional destabilization phenomena [16,17]. However, a well-known drawback of MEs is the high surfactant and/or co-surfactant composition, which may lead to skin irritation. NEs consisted of a lower amount of surfactant; however, its integrity is maintained consistently for a longer period than for micro-/macroemulsions, enhancing the product shelf life [10,14]. Among the ME and NE formulations, solubilized compounds in these systems are frequently lipophilic or hydrophobic in nature. Several investigations on peptide ME formulations reported the significant enhancement of the penetration of tetrapeptide (PKEK and GEKG) to the viable dermis layer over those in a standard cream [18]. However, a high amount of surfactant (25%) and oil content (5–65%) in the formula were highly likely to induce skin irritation [12,13,14]. A low surfactant ME development for poorly soluble drugs to avoid topical irritation was reported, however; over 16% of surfactants in the mixture had been used, and no development evidence of the factor affecting the low surfactant content in ME formation [19]. The superiority of MEs and NEs over conventional emulsions lies in their vast surface area, which allows for the transportation of hydrophilic active molecules via the polar heads of the surfactant film surrounding ultra-fine oil droplets. Additionally, they possess a greater ability to adhere to the skin, resulting in enhanced penetration through the membrane [15,20]. Therefore, this study pursued attempts to enhance dermal penetration and mitigate skin irritation caused by excess surfactants and oil phases in Tripeptide-3 micro- or nanoemulsion formulations. The development of the optimal delivery system was initiated from the investigation of relevant factors affecting the ME or NE formation using ternary phase diagrams, followed by using a design of experiment (DoE) program to find an optimized ME or NE formula for a routine anti-oil and shine facial essence. Caprylic/capric triglyceride (CCT), an effective emollient that is moisturizing, softening, and non-greasy, while creating a smooth and velvety texture ingredient, was selected as an oil phase for the developed MEs or NEs due to its various benefits for the skin [21]. In addition, surfactant, co-surfactant, and co-solvent compositions, and production protocols were demonstrated to obtain the isotropic MEs or NEs at a significantly lower content of surfactants in the system. Accordingly, various types of MEs or NEs generated in this study were compared for their physicochemical properties, stability, and in vitro skin permeation. Finally, the safety and efficacy of the derived optimized submicron-emulsion were confirmed in the volunteers who had oily facial skin using the before-and-after product application clinical trial.

## 2. Materials and Methods

### 2.1. Materials

PEG-40 hydrogenated castor oil (Cremophor^®^ RH40), and polyglyceryl-3 diisostearate (Lameform^®^ TGI) were purchased from BASF SE (Ludwigshafen, Germany). Sorbitan laurate (Span^®^ 20), and sorbitan monooleate (Span^®^ 80) were purchased from Guangdong Runhua Chemistry (Yingde, China); Caprylic/capric triglyceride (CCT or Lexol^®^ GT-865) was purchased from INOLEX Group (Philadelphia, PA, USA). Dipeptide diaminobutyroyl benzylamide diacetate (Tripeptide-3) CAS No. 823202-99-9, with purity of ≥95%, was purchased from Kangcare Biochemistry (Hong Kong, China). Tripeptide-3 standard, with purity of ≥98%, was purchased from Cayman Chemical, Ann Arbor, MI, USA. HPLC-grade acetonitrile, methanol, and deionized (D.I.) water were from RCI Labscan (Bangkok, Thailand). Trifluoroacetic acid (TFA) was purchased from Merck Schuchardt OHG (Hohenbrunn, Germany). All other chemicals were of the highest grade available.

### 2.2. Construction of Phase Diagram to Identify Factors Affecting Micro- or Nanoemulsion Formulation

A simple micro- or nanoemulsion production using water-titration technique was carried out using caprylic/capric triglyceride (CCT) as an oil phase. The optimization was first conducted by the selection of an appropriate hydrophilic–lipophilic balance (HLB) of non-ionic surfactant systems, followed by the fine-tuned selection of co-solvent and type of the co-surfactant for CCT oil phase and aqueous phase emulsification to obtain the isotropic (transparent and homogeneous) microemulsion. Oil and surfactant mixture (S_mix_) at various weight-ratios from 1:9, 2:8, 3:7, 4:6, 5:5, 6:4, 7:3, 8:2, to 9:1 were pre-mixed, and then water was gradually titrated into the test tube, followed by vortex mixing (15 s) after each drop, at an ambient temperature [22]. After being equilibrated, a clear liquid system was visually characterized as ME formation. Subsequently, the area of microemulsion formation was determined from pseudoternary phase diagrams [23,24], which were constructed using SigmaPlot^®^ trial version V.13 software by plotting the data points corresponding to the compositions (mass fractions) of the mixtures. Microemulsion formation extent (a single-phase area, %) in pseudoternary phase diagrams was calculated by ImageJ 1.54d public software (Wayne Rasband and Contributors, National Institutes of Health, Bethesda, MD, USA).

#### 2.2.1. Effects of HLB Value of Surfactant and Single-Tail Co-Surfactant System

The selection of surfactant systems based on the appropriate HLB value and surfactant combination on microemulsification was conducted. A hydrophilic surfactant, Cremophor^®^ RH40 (HLB of 14–16) [25], was mixed with a hydrophobic surfactant, either Span^®^ 20 (HLB of 8.6) or Span^®^ 80 (HLB of 4.3) [25], to obtain the predetermined HLBs of 7, 9, 11, and 13. The amount of each surfactant for the predetermined HLB_Mix_ was calculated using the alligation method, the so-called S_mix_ [26]. The S_mix_ was mixed with the CCT oil at the above ratios. Then, the aqueous phase was gradually added until a clear isotropic single phase was visually detected.

#### 2.2.2. Effect of Co-Solvent

Effects of co-solvent and ratios of water to co-solvent on microemulsion formation were determined. Isotropic area in ternary phase diagrams where the aqueous phase consisted of ethanol (EtOH) or propylene glycol (PG) at various ratios with D.I. water (1:1, 1:2, and 1:3) was determined by aqueous phase titration into the various ratios of the pre-mixed oil: S_mix_ (Section 2.2). The S_mix_ given the highest single-phase area from Section 2.2.1 was employed for the comparison of different water/co-solvent combination effect. Visual evaluation for the isotropy was performed for the ME extent in pseudoternary phase diagrams.

#### 2.2.3. Effect of Co-Surfactant Hydrocarbon Tails

**Figure 1:**
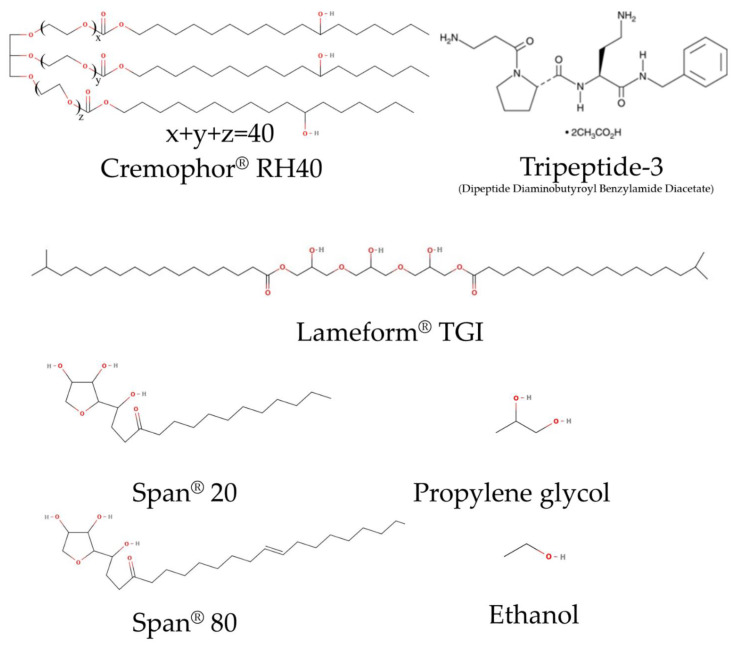
Illustration of Tripeptide-3, surfactant, and co-solvent structures used in the study.

Effect of co-surfactant structure was another factor influencing ME formation [25]. Geometric structures of surfactant molecules corresponding with critical packing parameter (CPP) were also compared for microemulsion formation and microscopic characters of the resulting microemulsion. A single-hydrocarbon-tail Span^®^20 (sorbitan monooleate), a surfactant with a single oleic hydrocarbon tail and Larmeform^®^ TGI (polyglyceral-3-diisostearate), a surfactant with double stearic hydrocarbon tails were compared for the ME formation when combined with the Cremophor^®^ RH40. Structures of compounds used in the optimization are depicted in Figure 1. S_mix_ from both surfactant systems was prepared to have the HLB of 13 and titrated with the water: PG at the ratio of 1:1. Little heat was applied to liquidify the surfactants before mixing. Various mixtures of oil to S_mix_ were prepared as aforementioned (Section 2.2); after the titration with a selected aqueous phase, the area of microemulsion formation and its microstructures were identified via visual evaluation of the isotropy in pseudoternary phase diagram and conductivity measurement. In the microemulsion area, four compositions were selected and characterized, representing the O/W, bicontinuous, W/O, and low surfactant MEs. The ME composition obtained from a low surfactant content in the pseudoternary phase diagram was further optimized using a full factorial design of experiment (DoE) for minimizing the surfactant used in the system.

#### 2.2.4. Design of Experiment Program (DoE) with Design Expert^®^ for Low Surfactant Micro- or Nanoemulsions

Based on the two-level full factorial experimental design (two-level FFD), the three factors were selected from the previous studies. These factors include HLB value (X_1_), propylene glycol content (X_2_), and oil content (X_3_). Each factor was divided into two levels, high and low, as shown in Table 1.

The S_mix_ containing Cremophor^®^ RH40 and Lameform^®^ TGI at 10% was used for all experiments in this section. Activation of ultra-fine emulsion formation by heating both oil and water phases up to 70 ± 2 °C [27], followed by hand mixing, could overcome the initial barrier of emulsification. Particle size (Y_1_) and percent transmittance (Y_2_) determined at 600 nm were the dependent responses. According to the FFD, 8 formulations were generated through a Design Expert^®^ program (Version 10.0.0, Stat-Ease, Minneapolis, MN, USA). The significant mathematic model (*p* < 0.05) with a high coefficient of determination (R^2^), as follows in Equation (1), was then generated based on the selected significant factors from the Pareto chart for describing the statistical correlation between factors and responses:

(1) $$\sqrt{Y_{1}}\mathrm{or}Y_{2}=\beta_{0}+\beta_{1\times 1}+\beta_{2\times 2}+\beta_{3}X_{3}+\beta_{12}X_{1}X_{2}+\beta_{13}X_{1}X_{3}+\beta_{23}X_{2}X_{3}+\beta_{123}X_{1}X_{2}X_{3}$$

where $\sqrt{Y_{1}}$ and Y_2_ represent particle size and percent transmittance, respectively, while X_1_, X_2_, and X_3_, at which the coefficient (β) of each variable is shown, are defined as the main effects of HLB value, propylene glycol content, and oil content, respectively. In addition, X_1_X_2_, X_1_X_3_, X_2_X_3_, and X_1_X_2_X_3_ represent the interaction effects of the factors. Moreover, the significance probability (*p*-value) of each regression coefficient was determined; *p* < 0.05 implies a significant term.

### 2.3. Characterization of Studied Colloidal Dispersion

The ME and NE compositions obtained from the aqueous phase titration (Section 2.2.3) and the DoE (Section 2.2.4) were prepared and characterized for their characteristics. Tripeptide-3 (0.012%) was dissolved in an aqueous phase before the submicron emulsion preparation. The MEs or NEs were maintained at room temperature for 24 h before being subjected to the characterization. All measurements were performed in triplicates at room temperature as follows:

#### 2.3.1. Polarized Light Microscopy

The samples were observed for their appearance visually and also microscopically using a polarized light microscope at room temperature. The samples were dropped onto a glass slide and covered with a glass cover slid before the observation under a microscope (Leica model PM RXP, New York, NY, USA) with a JVC Color Video camera at 10× magnification.

#### 2.3.2. Droplet Size and Distribution Determination

Droplet size and polydispersity index (PDI) of MEs or NEs were measured using a particle size analyzer at a 173° detector angle (Horiba S100, Kumamoto, Japan). The formulation was diluted with 18.2 MΩ/cm D.I. water at appropriate dilution for the transparent solution before measurement to avoid multiple scattering effects [28], the sample formulations were diluted to a concentration of 0.12 ppm, and stabilized for 2 h before the measurement.

#### 2.3.3. Turbidity Measurement

The turbidity of the formulated submicron emulsions was analyzed by measuring the absorbance at 600 nm. The percent transmittance of undiluted samples was recorded using UV–visible spectrophotometer (Shimazu 2600i, Kyoto, Japan).

#### 2.3.4. Electrical Conductivity

Measurement of electrical conductivity was performed for identification of ME or NE types. Electrical conductivity was determined with a conductivity meter (Eutech CON 150, Thermo Scientific, Singapore). The 0.9% saline solution was replaced with the D.I. water in each formulation. The saline solution and CCT oil were used as positive and negative controls, respectively.

#### 2.3.5. Viscosity Measurement

The viscosity of MEs or NEs was measured on a viscometer, Brookfield LVDV ΙΙΙ, equipped with cone and plate (Brookfield, Waukesha County, MA, USA) at a temperature of 30 ± 1 °C.

### 2.4. Determination of Tripeptide-3 with High-Performance Liquid Chromatography (HPLC)

HPLC-UV chromatography system (Shimazu LC-20AD, Kyoto, Japan) was employed for determination of Tripeptide-3. A reversed-phase C-18 column, (Eclipse XDB, 4.6 mm × 250 mm, 5 µm, Agilent, CA, USA) with a gradient-elution programmed analysis employed the mobile phase consisted of 0.1% trifluoroacetic acid and acetonitrile (ACN) as polar and non-polar mobile phases, respectively. The step gradient elution at a flow rate of 1 mL/min was performed by increasing the ACN from 8% to 16% for 21 min., then to 50% in 4 min, holding at this composition for 3 min, before decreasing ACN to 8% and holding at this composition, before a next injection that led to a total analytical time of 35 min per sample. The column temperature was maintained at 30 °C. A sample solution of 50 µL was injected into the column. The detection was carried out at 215 nm with a UV detector. The HPLC method validation was performed; the assay was linear (coefficient of determination, R^2^ > 0.999) in the Tripeptide-3 concentration range of 0.312–50.0 µg/mL with the lowest quantitation concentration of 312 ng/mL. An accuracy (% recovery) in the range of 90–106% was obtained with the intra- and inter-day precision (RSD) of less than 2.18%. The stability of the Tripeptide-3 in various pH of the colloidal dispersion and 24 h in autosampler was also determined.

### 2.5. Stability Tests of Colloidal Dispersion

#### 2.5.1. Stress Testing by Heating–Cooling Cycles

Effect of temperature variations on the stability of the submicron emulsions was investigated. Samples were stored between 4 and 40 °C, each for a period of 48 h. The heating–cooling cycle was repeated six times. The ME or NE formulations that passed the stress-induced instability such as turbidity, creaming, and phase separation were chosen and subjected to further freeze–thaw stress tests.

#### 2.5.2. Stress Testing by Freeze–Thaw Cycles

The formulations from the DoE were subjected to a freeze–thaw stress test between −20 and +25 °C with the storage period at each temperature for 48 h. Three freeze–thaw cycles were performed and the formulations that were stable to this stress were further characterized and preferred for the permeation and clinical application study.

#### 2.5.3. Stress Testing by Centrifugation

The formulated submicron emulsions from DoE were studied for their resistance to centrifugation. The formulations were subjected to centrifugation at 10,000 rpm (25,830× *g*) for 30 min (Sorvall ST16R, Thermo Fisher Scientific, Osterode am Harz, Germany). Phase separation, creaming, and turbidity (if any) were visually observed.

#### 2.5.4. pH Challenge Test

pH challenge test for the optimized MEs or NEs was modified from the previous study, including pH values of 4.5, 5.0, and 6.0 by adjusting the final formulation with citric acid [29]. Then, the stress stability tests (Section 2.5.1, Section 2.5.2 and Section 2.5.3) were repeated for assessment of physicochemical stability of the formulation.

### 2.6. Skin Permeation Study

Static Franz diffusion cells (Logan^®^ VCD-300, Logan Instruments, Somerset, NJ, USA) with Strat-M^®^ artificial membrane with a nominal surface area of 1.76 cm^2^ and a receiver compartment capacity of 12 mL were employed. The optimized formulation from the DoE referred to as the optimized formulation was selected to compare the Tripeptide-3 skin permeability with the MEs obtained from the pseudoternary phase diagram in Section 2.2.3. Effect of Co-Surfactant Hydrocarbon Tails and a coarse emulsion consisting of 10% oil, 5% S_mix_, and water phase. One milliliter of each formulation was added on the Strat-M^®^ in a donor chamber; the receiver was filled with the phosphate buffer, pH 7.4. The apparatus temperature was set at 32 °C. Samples (1 mL) in the receivers were withdrawn at 0, 2, 4, 8, and 12 h and replaced with an equal volume of fresh buffer. At the end of the study, the artificial membrane was removed and washed out for the remaining formulations, then cut into small pieces and suspended in 5.0 mL of water. Extraction for the absorbed peptide was performed by 15 min ultrasonication, followed by filtration with 0.22 µm nylon Acrodisc^®^ syringe filters (Pall Corporation Filtration and Separations Ltd., Bangkok, Thailand). The filtrate of 0.9 mL was mixed with 0.1 mL of paracetamol solution as an internal standard before being subjected to HPLC for the analysis. The study was performed in triplicates.

### 2.7. Skin Irritation Test and Efficacy Test of Tripeptide-3 Optimized Formulation in Human Volunteers

#### 2.7.1. Ethics Consideration

The study protocols for the skin irritation test and efficacy test were approved by the Human Research Ethics Committee of the Faculty of Pharmacy, Chiang Mai University, Thailand, before commencing the study (Cert.No. 002/2023/F).

#### 2.7.2. Human Volunteers

A total of 23 participants with oily skin aged between 20 to 40 years were recruited for skin irritation test. To identify volunteers with oily skin, a self-assessment questionnaire and measurement with a Sebumeter^®^ (SM810, CK, Courage and Khazaka Electronic GmbH, Cologne, Germany) were utilized for an evaluation. Volunteers with facial sebum content between 180–250 and 160–250 µg/cm^2^ for male and female subjects, respectively, were included in the study [30,31]. In addition, volunteers were required to be in good health, without any skin conditions, allergies to product components, undergoing medical treatment, using contraceptives, pregnant, or having skin issues affecting product usage.

#### 2.7.3. Skin Irritation Test

Skin irritation test was adapted from the OECD guideline, 2004 [32]. A Finn chamber^®^ (SmartPractice Europe GmbH, Greven, Germany) was used for evaluating the skin irritation of the volunteers. The optimized Tripeptide-3 product was added to the Finn chamber^®^ and compared with the positive control (2% *w*/*v* sodium lauryl sulfate) and negative control (deionized water). The Finn chamber^®^ on tape with tested samples was applied on the outer upper arm of the volunteers. The Finn chamber^®^ was then removed and observed for any skin reactions at 4, 24, 48, and 72 h. Primary dermal irritation index (PII) was calculated using this equation:

PII = [∑(erythema grade) + ∑(edema grade)]/(4 × N)(2)

where Σerythema grade is the sum of the erythema score after 4, 24, 48, and 72 h. The Σedema grade is the sum of the edema score after 4, 24, 48, and 72 h. N is the number of volunteers. PII values less than 0.5 indicate non-irritation, 0.5–2.0 indicates slight irritation, 2.1–5.0 indicates moderate irritation and more than 5.0 indicates severe irritation [32,33].

#### 2.7.4. Efficacy Test

For the efficacy study, healthy volunteers who met the inclusion criteria were included in a single group before and after study. The evaluation of the product’s efficacy spanned over 4 weeks. Prior to an assessment, volunteers were required to clean their faces for at least 3 h and acclimate their skin in a controlled-temperature room (25 °C) for a minimum of 30 min. Moisture content was measured on the forehead, cheek, and chin areas of volunteers using the Corneometer^®^ (CM825) (Courage and Khazaka Electronic GmbH, Cologne, Germany). The oiliness of the skin was measured on the forehead, nose, and chin areas of volunteers using the Sebumeter^®^ (SM815) (Courage and Khazaka Electronic GmbH, Cologne, Germany). The efficacy study was modified from the previous invasive botulinum toxin for the treatment of oily skin study. Measurements of sebum production were obtained using a Sebumeter^®^ at baseline and each follow-up visit [4]. The design of the trial was to measure the oil from the test strips tapped on three volunteer’s facial areas, before and after product application. The same person was his/her self-control, to prevent discrepancies between subjects [30,34]. However, a randomized placebo control could be used to exclude the effect of a formulation base. In addition, large and small facial pores and porphyrin content were evaluated using Visioface^®^ and Visiopore^®^, respectively. All volunteers used a standard facial wash product for cleaning their faces in the morning and evening. In the evening only, 10–15 drops of the optimized Tripeptide-3 product were applied to the entire facial area of the volunteers. The volunteers’ facial oiliness and moisture content were measured after using the assigned products for 2 and 4 weeks, respectively. The skin evaluation was compared before and after using the product for 2 and 4 weeks. The percent changes in skin moisture and oiliness were calculated from this equation:

The percent change = [(Before value − After value)/Before value] × 100(3)

where before and after values are the sebum content in the μg/cm^2^ unit.

### 2.8. Statistical Evaluation

Data were collected from three independent experiments and are shown as mean ± SD. Statistical analysis was performed using *t*-tests, ANOVA, and Tukey’s multiple comparison test, using SPSS, Version 19.0 (IBM^®^ SPSS Statistics, Armonk, NY, USA) and GraphPad Prism 8.0 (GraphPad Inc., San Diego, CA, USA). A *p*-value less than 0.05 was considered statistically significant.

## 3. Results & Discussion

### 3.1. Factors Affecting Micro- or Nanoemulsion Formulation

#### 3.1.1. Effects of Surfactant HLB Value and Single-Tail Co-Surfactant System

**Figure 2:**
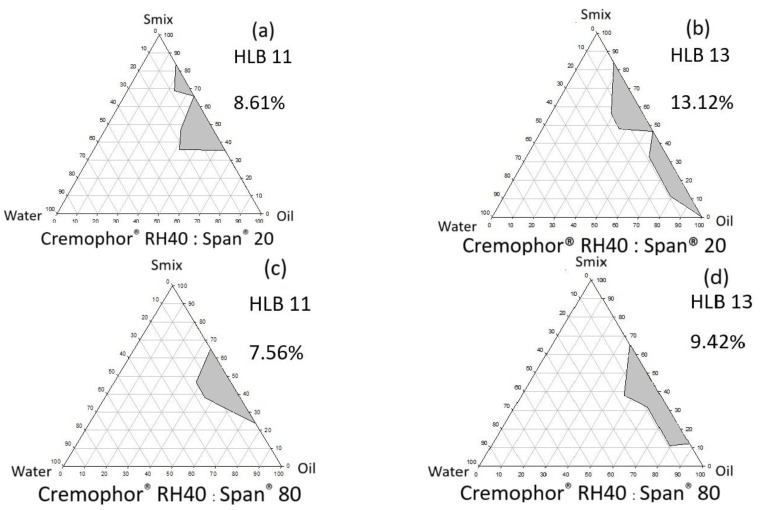
Pseudoternary phase diagrams and the microemulsified area (gray) of various CCT/water/S_mix_ as a function of the HLB 11 and 13. Area (%) of microemulsion formation of the Cremophor^®^ RH40 and Span^®^ 20 at HLB 11 (**a**) and HLB 13 (**b**) were compared to the area from the Cremophor^®^ RH40 and Span^®^ 80 at HLB 11 (**c**) and HLB 13 (**d**), respectively. The S_mix_ of Cremophor^®^ RH40 and Span^®^ 20 at HLB 13 were selected for further evaluation for the effect of co-solvent due to the highest microemulsion area was formed.

From the pseudoternary phase diagram in Figure 2, the microemulsion systems composed of the CCT oil:water:hydrophilic surfactant (Cremophor^®^ RH40) mixed with either Span^®^ 20 or Span^®^ 80, the hydrophobic single-tail co-surfactants, at the HLB values of 7 to 13 were firstly evaluated. The isotropic microemulsification (transparent) area in the diagrams (gray color) was formed only in oil as a continuous phase region even at a high HLB value (HLB 13). The surfactant compositions and the transparent area are shown in Table 2 and Figure 2. The largest transparent area (13.12%) was observed from the systems of HLB 13 of Cremophor^®^ RH 40:Span^®^ 20, while the area from the HLB 13 of the Cremophor^®^ RH 40:Span^®^ 80 system was only 9.42%. The longer hydrophobic chain of Span^®^ 80 produced a lesser microemulsification area than that from Span^®^ 20 when combined with the Cremophor^®^ RH 40. From the previous report, the Cremophor^®^ RH40:Span^®^ system was superior in terms of its microemulsion formation ability compared with a Tween^®^:Span^®^ system Weerapol et al., 2014 [25]. This observation resulted from the compatibility of the combined surfactant/co-surfactant system corresponding to the differences of the surfactant molecular geometry formation at a certain HLB. In the study, the isotropic microemulsion was not formed with the low HLB surfactants (HLBs 7 and 9). Singhan and Indranupakorn, 2015 [35] also found the lower microemulsion area in the pseudoternary phase diagram when the HLB values of Kolliphor^®^ and Span^®^ 80 were reduced. The S_mix_ of HLB 13 Cremophor^®^ RH40 and Span^®^ 20 was selected for further study due to the highest microemulsion area obtained. The results from these experiments revealed the crucial effects of HLB value and the molecular curvature of the surfactant system on the surface tension reduction between the oil and aqueous phases. Therefore, the critical packing parameter (CPP) should be considered when choosing the surfactants in ultra-fine colloidal formulation development.

#### 3.1.2. Effect of Co-Solvents

**Figure 3:**
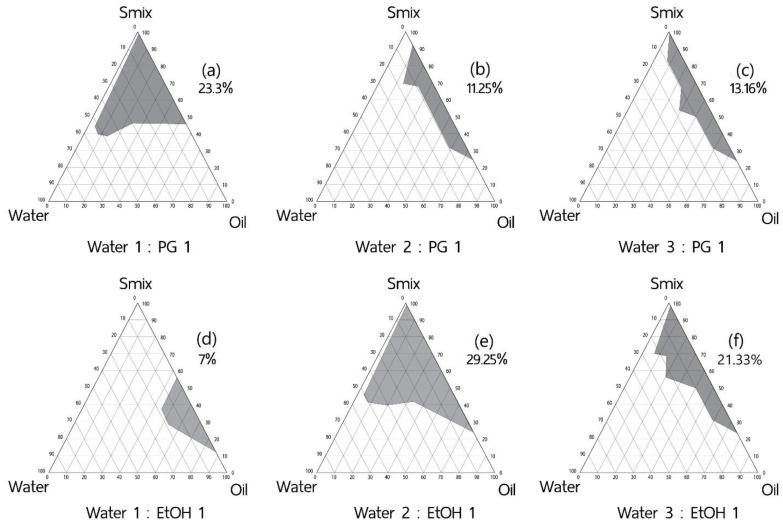
Pseudoternary phase diagrams and the microemulsified area of various CCT/S_mix_ Cremophor^®^ RH40 and Span^®^ 20 at HLB 13 as a function of an aqueous phase. Area (%) of microemulsion formation of the water-to-co-solvent ratios of 1:1, 2:1, and 3:1 were compared between two co-solvents, propylene glycol (PG) (**a**–**c**) and ethanol (EtOH) (**d**–**f**). Propylene glycol with a 1:1 water ratio (**a**) was selected for further study to assess the effect of double-tailed co-surfactants. This choice was made due to the high microemulsion area obtained and its superior skin compatibility compared to ethanol (EtOH).

The Cremophor^®^ RH40 and Span^®^ 20 at the HLB 13 system were selected for the study of a co-solvent effect. The optimal ratios of water to co-solvents using ethanol (EtOH) or propylene glycol (PG) contributed to a higher microemulsion area of 29.25% and 23.30% when 2:1 of water: ethanol, and 1:1 of water: PG were titrated into the oil and surfactant mixture (S_mix_) at various weight-ratios; Figure 3. The enhancement of microemulsion formation is in line with previous studies when isopropyl alcohol or butylene glycol were applied in an aqueous phase [18,35]. The mechanisms were due to the reduction of the intermolecular force between water molecules by the addition of less polar solvents to an aqueous phase, resulting in the cohesive force reduction of water and the improvement of the surfactant mixture performance on microemulsion formation [36]. To further develop the Tripeptide-3 formulation, the PG system was selected over ethanol because of the greater skin compatibility than ethanol when used as a skin permeation enhancer [37,38]. The water: PG system at a 1:1 ratio was selected to further evaluate the effects of the double-tailed co-surfactant on microemulsion formation.

#### 3.1.3. Effect of Co-Surfactant Hydrocarbon Tails

**Figure 4:**
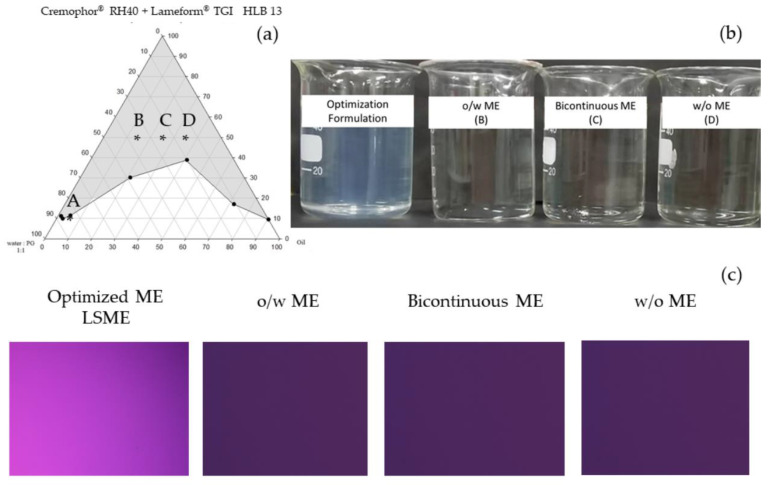
The effect Lameform^®^ TGI as a co-surfactant: (**a**) pseudoternary phase diagram and the microemulsified area of CCT/S_mix_ Cremophor^®^ RH40 and Lameform^®^ TGI at HLB 13 as a function of an aqueous phase (water:PG, 1:1); (**b**) MEs from point B, C, D, and the optimized formulation derived from point A; (**c**) polarized light microscope images show isotropic microstructure of all formulations. In the pseudoternary phase diagram (**a**), point A was selected for fine-tuned optimization using the design of experiment, while points B, C, and D were selected as the conventional MEs for comparison of skin permeability.

The highest part of the transparent area on a pseudoternary phase diagram was revealed when using Lameform^®^ TGI (polyglyceral-3-diisostrerate), a two-hydrocarbon-tails co-surfactant, substituted to Span^®^ 20 at HLB 13 in the water:PG (1:1) condition, 57.00% and 23.30%, respectively; Figure 3a and Figure 4a. By the addition of the Lameform^®^ TGI to the Cremophor^®^ RH40, the interfacial tension between the water phase (water:PG) and the CCT oil phase was greatly reduced with this new S_mix_ system. This result confirmed the improvement of S_mix_’s hydrophobic packing performance when these multi-tail surfactants were combined. Weerapol et al., 2012 studied the geometrical change and self-nanoemulsification to show the effect of the hydrophobic tails’ compatibility of the surfactant mixture on micelle formation [25]. Using a low HLB value double-tails co-surfactant contributed a significant effect on the geometrical shape of Cremophor^®^ RH40 together with the impact of PG as a co-solvent on reducing the cohesive force of water [36]. Consequently, Lameform^®^ TGI was selected as a co-surfactant to lower the amount of S_mix_ to 10% at point A, as shown in Figure 4a, over the Span^®^ 20 where 40% of the S_mix_ was required to form the ME. Although the low surfactant ME was achieved, concerning the existence of high PG content, more than 40% in an aqueous phase may be harmful for long-term facial application. From the pseudoternary phase diagram, the compositions obtained from points B, C, and D were prepared and characterized in comparison with the optimized formulation. Transparent MEs obtained when S_mix_ was 50% are illustrated as shown in Figure 4b. The microstructures of the prepared MEs were the O/W (B), bicontinuous (C), and W/O (D) MEs, specified from the electrical conductivity values of the continuous phase of 208.53 ± 0.7, 81.92 ± 0.1, and 28.40 ± 0.1 μS/cm, compared with the optimized formulation of 3663.58 ± 5.8 μS/cm from the DoE, respectively (Table 3). In addition, these samples were examined by a polarized light microscope and did not observe birefringent or colorful images, which indicates no formation of the liquid crystalline phase at room temperature Figure 4c. The fine-tuned optimization for a lower amount of PG was discussed in detail in the DoE approach.

#### 3.1.4. Design of Experiment Program (DoE) with Design Expert^®^ for Low Surfactant Micro- or Nanoemulsions

**Figure 5:**
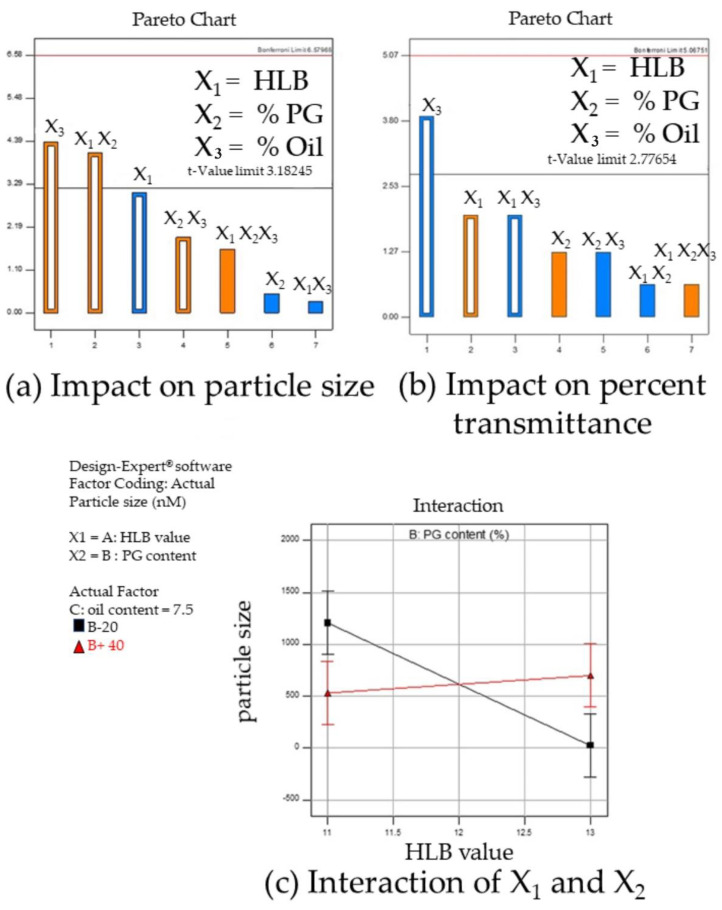
The Pareto chart obtained from the Design Expert^®^ program demonstrates a statistically significant variable factor (above t-value limit) impact on responses: (**a**) indicates X_3_ (oil content) and interaction between X_1_ (HLB value) and X_2_ (PG content) exhibited statistically significant impact on particle sizes of ME; (**b**) shows statistically significant effect of oil content on percent transmittance of ME; and (**c**) demonstrates X_1_ (HLB value) and X_2_ (PG content) interaction effect on particle sizes of ME from DoE experiment when oil content was constant; the lowest particle size (■ black line) was formed when HLB value of S_mix_ and % PG were 13% and 20% in the formulation.

The DoE was used for the fine-tuning optimization to obtain a transparent and stable colloidal dispersion requiring a low amount of surfactants. The preliminary excipient screening at an ambient condition revealed the effects of high and low levels of critical components such as the HLB value of the S_mix_ and co-solvent. A lower amount of co-solvent likely encountered a reduced transparency of emulsion due to the kinetic barrier of water molecules [27]. The desirable formulation attained by preparing the submicron micro- or nano-emulsions with DoE conditions is referred to as the optimized formulation. Eight formulations based on a two-level full factorial design (FFD) covering the high and low levels of three factors, together with the responses and residues used to measure the error in the models, are shown in Table 4. At 10% S_mix_, three of the eight formulations exhibited a transparent/translucent emulsion with droplet diameters less than 100 nm. Formulation 3 (F3) possessed the translucent dispersion, having the mean droplet size of 81.2 ± 11.5 nm, and was highly likely to form NEs. Smaller droplet sizes of the transparent dispersion were obtained from F4 (25.7 ± 1.20 nm, PDI 0.237 ± 0.129) and F5 (28.3 ± 1.80 nm, PDI 0.147 ± 0.101) with a high percent transmittance, when the HLB was 13 and oil content was 5%. The difference was only the PG content of 20 and 40%, respectively (Table 4). Herein, the Pareto charts (Figure 5a,b), which are a graphical tool used for prioritizing the factors that have the most significant impact on the responses in terms of the absolute t-value, were generated for each response [39]. To classify the significant levels of the effects, the Bonferroni limit and t-value limit, which are the threshold, were considered. The t-values of effects above the t-value limit were considered as moderately significant effects, while the values above the Bonferroni limit were considered as strongly significant effects of which the significant effects were subsequently included in the final model. Conversely, the t-values of the effects below the t-limit were insignificant terms [40]. According to the Pareto chart (Figure 5a), significant positive values of the oil content (X_3_) (*p* = 0.0224) together with the interaction between the HLB and PG content (X_1_X_2_) (*p* = 0.0266) were the significant terms for droplet size discrepancy. The R^2^ greater than 0.80 can be accepted to offer a reliable model [41]. The empirical mathematic equation was then generated with a high coefficient of determination R^2^ = 0.94, indicating an approximately 94% accuracy of the study model (*p* = 0.0337). The inclusion of non-significant terms including X_1_ (*p* = 0.0548) and X_2_X_3_ (*p* = 0.1482) could enhance the accuracy of the final model:

(4) $$\sqrt{Y_{2}}=616.38-253.65X_{1}+360.48X_{3}+337.60X_{1}X_{2}+160.27X_{2}X_{3}$$

Following the correlation, it could be interpreted that a growing up of the particle size occurred when the oil content increased together with lower values of HLB. The higher oil content in an emulsion system generally leads to larger droplet sizes. This phenomenon can be attributed to the fact that, as oil is added to the system, the continuous phase becomes more saturated with oil droplets, leading to coalescence and the formation of larger droplets [42]. In addition, the significant interaction effect between X_1_ and X_2_ referring to the HLB value and PG amounts on the particle size was observed when the oil content was at a constant level (Figure 5c). At the condition of a low level of PG (20%) and a high level of the HLB value, the system containing the smallest particle sizes could be obtained. The integration of a low concentration of PG with surfactants possessing high HLB values plays a crucial role in minimizing the particle size of the system. This significant interaction not only reduces the interfacial tension but also optimizes the surfactant layer’s curvature around the oil droplets, thus facilitating the formation of emulsions with the smallest particle sizes. Aside from the particle size, the mathematic equation illustrating the significant correlation between factors and the system’s turbidity response (*p* = 0.039) in terms of the percent transmittance was generated, according to the Pareto chart (Figure 5b), where only the main effect of the oil content (X_3_) (*p* = 0.0176) was significant. After the inclusion of X_1_ (*p* = 0.1183) and the interaction term of X_1_X_3_ (*p* = 0.1183), the model’s R^2^ of 0.85, which is above the lower limits 0.80 [39], was shown with an approximately 85%accuracy of the study model:

% Transmittance = 26.164 + 13.321X_1_ − 26.143X_3_ − 13.32X_1_X_3_ (5)

The equation represents a negative value of the oil content (X_3_) indicating that the oil content has an inverse effect on the transparency of the formulation. The transparency of an emulsion is significantly influenced by the size and distribution of its dispersed droplets. When the oil content in the system is reduced, the concentration and, possibly, the size of the oil droplets within the continuous phase decrease. This reduction in size and concentration decreases the overall scattering of light, leading to increased transparency [42].

### 3.2. Stability Tests of Colloidal Dispersion from DoE

**Figure 6:**
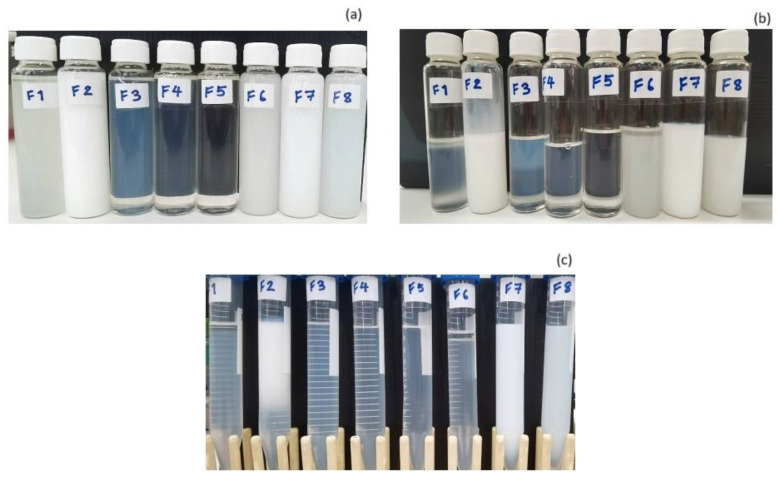
Stress stability studies of micro- and nanoemulsion preparations from the DoE study (**a**) overnight after preparation; (**b**) 6 cycles of heating and cooling between 4 and 40 °C, each for a period of 48 h, and (**c**) after 30 min of 10,000 rpm (25,830× *g*) centrifugation.

**Figure 7:**
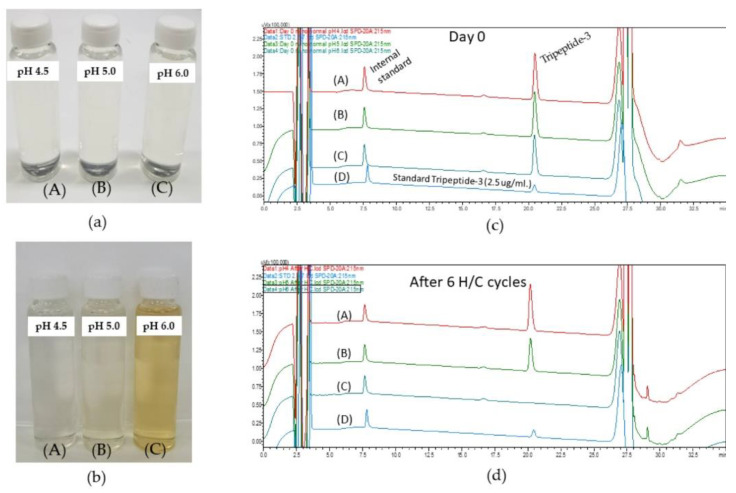
Physical and chemical stability of the optimized formulation with adjusted pH of 4.5, 5.0, and 6.0 at Day 0 (**a**). After six heating/cooling cycles, pH 5.0 shows slight yellow (B), while pH 6.0 turns to full yellow (C). No color change occurred in pH 4.5 formulation (A) (**b**). The HPLC chromatograms of Tripeptide-3 revealed the chemical stability of the Tripeptide-3 in the optimized formulation at different pH: (**c**) represents Day 0, (**d**) represents after 6 H/C cycle of optimized formulations, with (A) in pH 4.5, (B) in pH 5.0, (C) in pH 6.0, and (D) a standard Tripeptide-3. Paracetamol, an internal standard, and the Tripeptide-3 peaks were eluted at retention times of 7.6 min and 20.5 min., respectively.

All formulations from the DoE were subjected to stress stability tests (Figure 6a). After passing through six cycles of heating and cooling (H/C), only F4 and F5 remained with an isotropic characteristic (Figure 6b). In another stress test using centrifugation at 10,000 rpm (25,830× *g*) for 30 min, no phase separation or coalescence, a sign of macroscopic stability, was observed in the F4 and F5 formulations; other formulations exhibited a greater turbidity or phase separation as shown in Figure 6c. Nevertheless, the particle size measurement analysis showed that the particle sizes of the F4 and F5 formulations decreased after six cycles of heating/cooling (Table 5). Only F5 exhibited a statistically significant change in particle size. Consequently, the final composition of F4 was selected for the Tripeptide-3 entrapment. The Tripeptide-3–loaded formulation (F4) revealed a homogenous, bluish translucent morphology with low viscosity. In comparison with the O/W, bicontinuous, and W/O MEs (Figure 4b), the conductivity (Table 3), microscopic polarized light image (Figure 4c), and water dilution ability (100 times) (Table 6) of the selected F4 were distinct from the MEs, indicating the formulation is an oil-in-water-type nanoemulsion. In addition, the optimized NEs made up only a 2:1 ratio of the surfactant to oil, which is much lower than the ratios normally used in the ME formation [43]. The optimization of the pH in the system revealed the changes after the accelerated stability determination (six cycles heating/cooling between 4 and 40 °C, and three cycles freeze/thaw between −20 and +25 °C with the storage period at each temperature for 48 h), indicating the reduced thermodynamic stability of the Tripeptide-3 NEs. The instability was pronounced in pH 5.0 and 6.0; Figure 7 and Table 6. However, the particle size of the pH 4.5 NEs stored at room temperature for 180 days remained the same, assuring the kinetic stability of the optimized NEs; Table 6. Unlike the ME formation, energy, either high or low, is required for the NE formation. The phase transition temperature, microemulsion dilution, solvent displacement/diffusion and spontaneous emulsification methods use less energy for the NE preparation [44]. In this study, the low-energy method using heat and stirring was chosen to develop NEs. The preparation of NEs and optimization studies involved applying heat (70 ± 2 °C) to both the oil and water phases before mixing. This process reduced the free energy of the system, promoting the formation of kinetically stable NEs. The optimal compositions of excipients and the internal radii of <100 nm (particle size~25–28 nm) which allow the Brownian motion of the NEs could be the predominant reasons for keeping the system stable and avoiding physical instabilities indicated by flocculation, sedimentation, and creaming [27,45].

In terms of the chemical stability of Tripeptide-3 NE, the amount of Tripeptide-3 in NE remained stable at pH 4.5 after the six-cycle H/C stress test (Figure 7a,b). The gradient HPLC-UV analysis determined the peptide and paracetamol (I.S.) peaks at 20.5 min and 7.6 min, respectively. There were changes in the mobile phase compositions during the gradient-programmed analysis; the early eluted (solvent front) and the later peaks of less polar compounds and the abrupt change in mobile phase compositions at 2 and 27 min were observed, which is not an unusual observation when the UV detection was set at 215 nm. These results indicated that the developed HPLC analysis method was suitable for the Tripeptide-3 analysis.

MEs and NEs are very similar in many characteristics and it is difficult to give a clear-cut explanation of the characterization; a detailed examination of the terminology, differences, and similarities of NEs and MEs is provided by McClements [27], and recently reviewed by Nastiti et al. [43]. When considering the primary factors for identifying the optimized dispersion as NEs, it is evident that the diluted ability, kinetic stability, and reduced thermodynamic stability of the resultant system, along with the low-energy input necessary for transparent/translucent submicron emulsion formation in this investigation, collectively emphasize this determination.

### 3.3. Skin Permeation of Tripeptide-3 Micro- and Nano-Formulations

**Figure 8:**
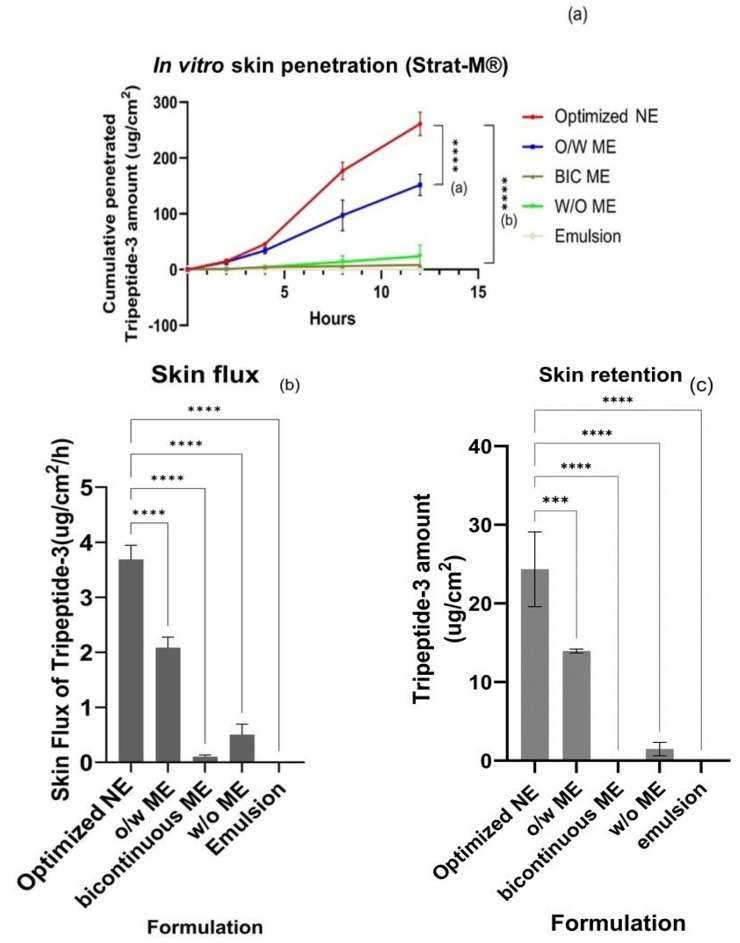
Comparative amounts of Tripeptide-3 in different formulations across the membrane and accumulated in the receivers from Franz diffusion study (**a**), and skin flux determined from each formulation showing statistically significant higher percutaneous absorption rate of Tripeptide-3 optimized nanoemulsion formulation compared with the MEs and emulsion (**b**); Tripeptide-3 amount per cm^2^ membrane retained in the skin from each formulation after 12 h of the permeation study (**c**). Each value represents the mean ± SD (n = 3). *** *p* < 0.001; **** *p* < 0.0001.

Strat-M^®^ is a widely used artificial skin for permeation study. It is an artificial membrane envisioned as an alternative to animal and human skin with similar factors as skin layers to mimic human skin. This multi-layer artificial membrane possesses a tight top layer coated with a lipid blend resembling the lipid chemistry of the human stratum corneum (SC) and a porous lower layer resembling the epidermis and dermis layers. The validation against EpiSkin^®^ RHE and human excised skin revealed closer permeability results to those from the excised human skin. Therefore, this study decided to use Strat-M^®^ instead of human skin or animal skin for the skin permeation study [46,47]. The difference in the cumulative amount and skin flux of Tripeptide-3 test formulations for 12 h was shown in Figure 8a,b. The optimized Tripeptide-3 NE showed a statistically significant higher cumulative amount and skin flux over other test formulations, especially the simple O/W emulsion of Tripeptide-3, which had not been detected in the receiver at all studied time points. The ultra-high surface area of optimized Tripeptide-3 NE droplets and lower viscosity when using lower surfactant percentages may be the critical factors for improving skin permeability compared with other high S_mix_ in MEs [41]. NEs with lipidic structures are considered ultra-flexible and likely to form any shape during transportation through the skin. With the droplet size below 50 nm, the higher penetration to the deeper layer of the skin by passive diffusion and appendage shunt pathways such as hair follicles and sweat glands was enhanced [47,48,49]. The content of propylene glycol (PG) in the developed systems in the optimized NE, O/W ME, bicontinuous ME, and W/O ME were 20%, 22.5%, 12.5%, and 7.5%, respectively. Having ultra-fine droplets and a high penetration enhancer (PG) in the NE system, membrane fluidity and the interaction of the carriers with the skin were promoted, allowing the exchange between the outermost layers of the stratum corneum and the carriers [49]. However, the Strat-M^®^ membrane only differentiates the penetration ability by passive diffusion among different formulations.

A skin retention experiment showed the highest amount of Tripeptide-3 from the optimized NE, statistically significantly higher than other test formulations; Figure 8c. The lower propylene glycol and bicontinuous ME and W/O ME, and the absence in the emulsion may limit the permeation performance of these formulations. From our results, the transdermal performance of the optimized NE was less than 1% of the given amount, but rather localized in the skin. The fluidic microstructure, high solubilizing capacity, and excellent skin affinity from the dermatopharmacokinetic analysis of the nanoemulsion components across the skin depth were reported for the greater skin permeation of the NEs [49]. Therefore, the delivery of Tripeptide-3 NE to deeper skin layers as the target sites is logical for topical application [50].

### 3.4. Clinical Evaluation

#### 3.4.1. Skin Irritation Test in Volunteers

**Figure 9:**
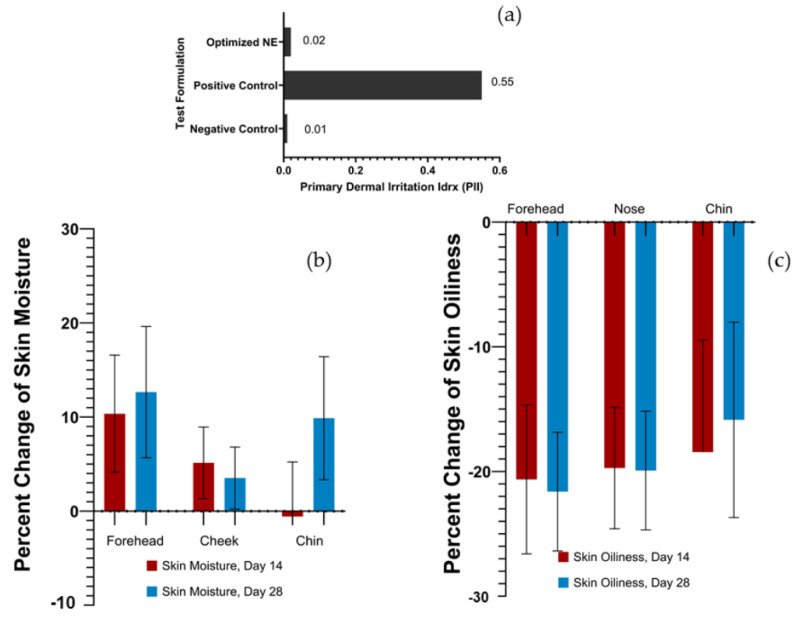
Dermal irritancy and efficacy of Tripeptide-3 nanoemulsion from clinical evaluation: (**a**) skin irritation comparison (PII values) from Tripeptide NE, positive and negative controls, (**b**) skin moisture improvement when applied on facial zones, and (**c**) reduction of skin oiliness comparison between different facial zones on day 14 and day 28. Mean ± SD, n = 23 (paired *t*-test, *p* < 0.05, on day 0 vs. day 14 and day 18).

The skin irritation of the Tripeptide-3 NE product was evaluated using a patch test (Finn^®^ chamber) in comparison to SLS (positive control) and DI water (negative control) in 23 volunteers. The results showed that the Tripeptide-3 NE product did not induce erythema and edema on the tested areas after 4, 24, 48, and 72 h. In contrast, SLS generated edema on the upper arm of volunteers after 4, 24, 48, and 72 h. The PII value of the Tripeptide-3 NE product was 0.02, indicating no skin irritation, similar to the negative control (the PII value of 0.01). Conversely, the PII value of SLS was 0.55, indicating slight irritation. These results are shown in Figure 9a, indicating that the Tripeptide-3 NE product is safe for further use in the efficacy test.

#### 3.4.2. Efficacy Evaluation of the Tripeptide-3 NE Product in Volunteers

Twenty-three healthy volunteers aged between 20–40 years, who did not experience irritation from the product, were enrolled in the efficacy evaluation. Oiliness and moisture of the forehead, nose, and chin were evaluated as the primary endpoints using Sebumeter^®^ and Corneometer^®^, comparing measurements taken on day 0, day 14, and day 28. In addition, large and small facial pores and the porphyrin amount, measured by Visioface^®^ and Visiopore^®^, respectively, were set as the secondary endpoints. The product was applied every evening for 4 weeks. The results demonstrated that the skin moisture of the volunteers increased after using the product for 14 and 28 days, especially on the forehead and cheek areas, whereas the skin moisture of the chin increased after using the product for 28 days. The percent change of skin moisture in the forehead, cheek, and chin after using the product for 28 days were 12.65 ± 6.98%, 3.52 ± 3.29%, and 9.88 ± 6.54%, respectively; Figure 9b. These findings indicated that the Tripeptide-3 NE product helped to improve skin hydration. A statistically significant reduction in sebum production compared to the baseline was observed on the forehead and nose with percentages of 21.61 ± 4.76% and 19.92 ± 4.76%, but the reduction was observed but not statistically different for the chin (15.85 ± 7.83%) after using the product for 28 days. However, the product demonstrated a reduction in sebum production in all tested areas; Figure 9c. Five of the male volunteers improved skin refining by reducing large and small facial pores after using the product for 28 days, resulting in a 45% reduction. Moreover, no difference was observed in the amount of porphyrin among all volunteers.

The optimized Tripeptide-3 NEs effectively reduced facial oil and increased skin moisture within 14 days when applied only once a day in the evening. Previous research has reported that palmitoyl Tripeptide-3, a more lipophilic analog, can mimic the thrombospondin 1 Tripeptide sequence and collagen synthesis via TGF-β, making it suitable for use as an anti-wrinkle, firming agent, and a skin moisturizer in cosmeceutical products [51]. To date, a clinical study of a Tripeptide-3 topical formulation for oil control has not been reported. The compilation of topical peptides for anti-wrinkle and/or anti-aging was demonstrated by Gorouhi and Maibach. In comparison with the 10% Argireline cream, the 4% Tripeptide-3 (Syn-ake^®^) cream had surpassed it in anti-aging efficacy in the before/after clinical trial [52].

In this study, not all volunteers exhibited skin-refining improvement. The great variation is impacted by various factors such as genetics and lifestyle that influence facial pore enlargement. In addition, the existing collagen density on the face compromised the refining effect [53]. The optimized NEs from this study did not show an advantage on porphyrin reduction. Porphyrin is a compound derived from Cutibacterium acnes. The presence of porphyrin is related to sebaceous gland activity and sebum content [54,55]. High sebaceous gland activity or high sebum secretion frequently causes a high amount of porphyrin. Nonetheless, the porphyrin amount depends on bacteria metabolism rather than facial oil content [56].

## 4. Conclusions

The nanoemulsion, containing a low surfactant content for the delivery of Tripeptide-3, was successfully developed. The resultant translucent liquid formulation demonstrated a high physicochemical stability after being stored for 180 days. The oil droplets exhibited a uniform distribution, with sizes ranging approximately between 20 and 30 nm. A strategic variation in the development of this ultra-fine colloidal dispersion was carried out using a pseudoternary phase diagram plot, aimed at identifying highly impactful factors for microemulsion formation. By the application of the design of experiments (DoE) approach, the kinetically stable nanoemulsions were obtained. The nanoemulsion was formed utilizing a low-energy emulsification method, employing heat to emulsify the CCT oil and water phases. This process required only 10% of Cremophore^®^ RH40 as a surface-active agent, along with polyglyceral-3-diisostrerate as a co-surfactant, and propylene glycol as a co-solvent. The stabilization of the Tripeptide-3 content and the nanosized droplets was achieved at a pH of 4.5 within the formulation. The resulting nanosized droplets possessed a significant interfacial surface area and exhibited high flexibility, thereby enhancing the penetration and retention of Tripeptide-3 in the deeper layers of the Strat-M^®^ membrane. This effect was notably superior to that observed with high surfactant microemulsions and coarse emulsions. The formulation’s unique properties allow for the utilization of a sufficiently low surfactant content, ensuring product safety. Moreover, the efficacy of the final product in reducing oiliness was demonstrated in a clinical study in human volunteers with oily facial skin. A reduction of approximately 20% in sebum production was observed after 28 days of application. Furthermore, the NE formulation was found to enhance skin moisture. In summary, nanoemulsions with a low surfactant content present a promising avenue as an effective colloidal carrier for delivering a hydrophilic substance to the skin.

## Figures and Tables

**Table 1:** Ranges of excipient content in two-level full factorial design.

Factors		Level	
		Low Level	High Level
X _1_	HLB value	11	13
X _2_	Propylene glycol content	20%	40%
X _3_	Oil content	5%	10%

**Table 2:** Compositions of surfactant mixture (S_mix_) were calculated using an alligation method for preparation S_mix_ at HLB 7, 9, 11, and 13. Percentages of microemulsion area on pseudoternary phase diagram were demonstrated, resulting in the highest area from Cremophor^®^ RH40:Span^®^ 20 system that was selected for the study of co-solvent effect.

HLB	Surfactant Mixing Ratio (% *w*/*w*)		ME Area (%)
	Cremophor^®^ RH40	Span^®^ 80	
7	25.2	74.8	0
9	43.9	56.1	0
11	62.6	37.4	7.56
13	81.3	18.7	9.42
HLB	Cremophor^®^ RH40	Span^®^ 20	
7	N/A	N/A	N/A ^1^
9	6.3	93.8	0
11	37.5	62.5	8.61
13	68.8	31.3	13.12

Note ^1^: N/A = not available (S_mix_ at the HLB 7 could not be prepared).

**Table 3:** Electrical conductivity measurement of optimized NE and ME for determination of continuous phase.

Electrical Conductivity (μS/cm)			
Optimized NE	O/W ME	Bicontinuous ME	W/O ME
3663.58 ± 5.8	208.53 ± 0.7	81.92 ± 0.1	28.40 ± 0.1

**Table 4:** DoE and responses from variable factors of two-level full factorial experimental design (FFD).

		Factor 1	Factor 2	Factor 3	Response 1	Residuals of Response 1	Response 2	Residuals of Response 2
Std	Run (F)	A:HLB Value	B:PG Content (%)	C:Oil Content (%)	Particle Size (nm)		Turbidity (% Transmittance)	
7	1	11	40	10	901.9 ± 911.20	−353.35	0.0 ± 0.01	0.000
5	2	11	20	10	1608.6 ± 1040.90	353.35	0.0 ± 0.01	0.000
3	3	11	40	5	81.2 ± 11.50	−12.55	51.2 ± 0.01	5.310
2	4	13	20	5	25.7 ± 1.20	−1.3	70.6 ± 0.58	−5.565
4	5	13	40	5	28.3 ± 1.80	1.3	87.3 ± 0.01	0.565
8	6	13	40	10	1290.6 ± 1098.20	201.1	0.0 ± 0.00	−0.003
6	7	13	40	10	106.3 ± 3.90	12.55	0.0 ± 0.00	−5.310
1	8	11	20	5	888.4 ± 809.10	−201.1	0.1 ± 0.01	0.003

**Table 5:** Stability of the selected formulations obtained from DoE.

Formulation	Particle Size (nm) Day 0	Percent Transmittance (%)	Centrifuge at 10,000 rpm 15 min	Heating/Cooling 6 Cycles	Particle Size (nm) After H/C
3	81.2 ± 11.50	51.2 ± 0.01	Stable	x	N/A
4	25.7 ± 1.20	70.6 ± 0.58	Stable	Stable	22.5 ± 1.80
5	28.3 ± 1.80	87.3 ± 0.01	Stable	Stable	23.58 ± 1.04 *

Note: Stable = no phase separation, x = phase separation, N/A = data not available due to the unstable formula. * *p* < 0.05 (particle size on day 0 vs. after H/C); the samples were diluted 100 times with D.I. water for particle size and PDI measurement.

**Table 6:** Particle size and PDI measurement (diluted 100 times with D.I. water) of the optimized formulations with adjusted pH to 4.5, 5.0, and 6.0, at the end of each accelerated test and after 180 days of storage at room temperature.

Formulation 4	Particle Size Measurement			
	Day 0 Size nm (PDI)	After 6 H/C Cycles Size nm (PDI)	After 3 F/T Cycles Size nm (PDI)	Day 180 Size nm (PDI)
pH 4.5	28.1 ± 0.7 (0.27 ± 0.010)	26.7± 0.3 * (0.149 ± 0.028) * *p* = 0.0334	31.5 ± 0.3 ** (0.262 ± 0.014) ** *p* = 0.0015	29.1± 0.4 (0.075 ± 0.038) *p* = 0.0599 ns
pH 5.0	23.9 ± 0.3 ^###^ (0.152 ± 0.002) ^###^ *p* = 0.0007 vs. pH 4.5)	30.1 ± 0.0 **** (0.258 ± 0.016) **** *p* < 0.0001 vs. day 0	26.1 ± 0.2 *** (0.154 ± 0.01) *** *p* = 0.0005 vs. day 0	N/A
pH 6.0	24.3 ± 0.1 ^###^ (0.267 ± 0.032) ^###^ *p* = 0.0007 vs. pH 4.5	25.4± 0.5 * (0.220 ± 0.037) * *p* = 0.0202 vs. day 0	28.0 ± 0.3 *** (0.278 ± 0.015) **** *p* = 0.0001 vs. day 0	N/A

Note 2: N/A = not available. (* *p* < 0.05, ** *p* < 0.005, *** *p* ≤ 0.0005, **** *p* < 0.0001), ^###^ compared with pH 4.5, ns, not significant).

## Data Availability

Raw data availability was archived, and the materials are available upon request.
